# The role of gut microbiome in antimicrobial resistance transmission between companion animals and livestock: mechanisms, drivers, and One Health implications

**DOI:** 10.3389/fmicb.2026.1872946

**Published:** 2026-06-29

**Authors:** Jianping Guo, Huiting Song, Zhaoyan Xi, Wei Geng, Fangfang Wang

**Affiliations:** 1 Department of Respiratory and Critical Care Medicine, The Affiliated Hospital of Qingdao University, Qingdao, China; 2 Qingdao Women and Children’s Hospital, Qingdao University, Qingdao, China; 3 Department of Ultrasound Diagnosis, Qingdao Special Servicemen Recuperation Center of PLA Navy, Qingdao, China; 4 Department of Neurology, The Affiliated Hospital of Qingdao University, Qingdao, China

**Keywords:** antimicrobial resistance, companion animals, gut microbiome, livestock, One Health

## Abstract

Antimicrobial resistance (AMR) poses a critical global public health challenge, with animal gut microbiomes serving as significant reservoirs and transmission hubs for antimicrobial resistance genes (ARGs). This review synthesizes current knowledge on the central role of gut microbiomes in companion animals and livestock in facilitating AMR dissemination. It examines key mechanisms that enable horizontal gene transfer within intestinal ecosystems: conjugation, transduction, and transformation. It also highlights how co-selection by heavy metals, disinfectants, and other non-antibiotic agents sustains resistance even without direct antibiotic use. The review analyzes major drivers of AMR, including antimicrobial usage, husbandry practices, and environmental pressures. It critically evaluates microbiome-based interventions such as probiotics, postbiotics, and fecal microbiota transplantation. A distinctive contribution is the integration of these elements into a network-centric One Health framework that explicitly maps cross-species transmission pathways from livestock and companion animals to humans via direct contact, food chains, and environmental dissemination. By moving beyond descriptive cataloging to provide a mechanistic and ecological synthesis, this review aims to guide the development of targeted, microbiome-informed intervention and surveillance strategies.

## Introduction

The escalating global crisis of antimicrobial resistance (AMR) represents a formidable threat to the efficacy of infectious disease treatments in both human and veterinary medicine. This challenge is intrinsically linked to the complex microbial ecosystems residing within the gastrointestinal tracts of animals. In particular, companion animals and livestock serve as critical reservoirs and amplifiers of antibiotic resistance genes (ARGs). The gut microbiome functions not only as a natural repository for diverse ARGs but also as a hotspot for horizontal gene transfer (HGT), facilitating the dissemination of resistance determinants among bacterial populations (McInnes et al., 2020). While previous reviews have catalogd ARGs in animal microbiomes or described horizontal gene transfer (HGT) mechanisms, critical knowledge gaps remain. First, the relative contributions of different HGT mechanisms (conjugation, transduction, transformation) to *in vivo* ARG dissemination under real-world farming conditions are poorly quantified (McInnes et al., 2020; Borodovich et al., 2022). Second, direct antibiotic selection and indirect co-selection pressures both drive resistance enrichment in animal guts. However, their interplay is not systematically understood (Nemathaga et al., 2023). Third, a conceptual framework that integrates the gut microbiome as an ecological network, rather than a passive carrier, to explain cross-species AMR transmission under the One Health paradigm is lacking (Scicchitano et al., 2023; Tian et al., 2025). This review addresses these gaps by adopting a network-centric ecological perspective. We synthesize current knowledge to argue that the animal gut microbiome functions as a crucible for AMR emergence and a hub for its inter-host propagation. The intimate cohabitation between humans and companion animals, such as dogs, creates a unique interface for microbial exchange. Metagenomic analyses have demonstrated a strong correlation in the gut resistome profiles between owned dogs and their human owners, with compositions of ARGs and mobile genetic elements (MGEs) being significantly more similar within households than with unrelated individuals (Zhao et al., 2022). This shared resistome is further evidenced by the finding that dog owners exhibit a significantly higher abundance of specific aminoglycoside and tetracycline resistance genes compared to non-dog owners. This highlights the bidirectional flow of resistance determinants at the human-animal interface (Cui et al., 2026). A critical examination of the literature reveals significant methodological and conceptual limitations. For instance, metagenomic analyses have demonstrated strong correlations in gut resistome profiles between dogs and their owners (Zhao et al., 2022). However, these studies are inherently associative. They cannot establish causality or directionality of transmission. Metagenomic assembly is also challenged by strain-level resolution and the frequent inability to link ARGs to specific mobile genetic elements (MGEs) or viable bacterial hosts (McInnes et al., 2020). These inconsistencies underscore the need for mechanistic, hypothesis-driven research. Throughout this review, we explicitly distinguish between (i) correlative associations, (ii) inferred ecological linkages, (iii) confirmed transmission events, and (iv) causal pathways demonstrated by longitudinal or experimental studies. Where the literature provides only correlative evidence, we note this limitation.

In livestock production systems, the extensive and often subtherapeutic usage of antimicrobials exerts immense selective pressure, driving the evolution and enrichment of resistant bacteria within the animal gut. The swine gut microbiome, for instance, has been characterized as a highly diverse and relatively conserved reservoir of ARGs. It is frequently associated with a wide range of MGEs, including integrative conjugate elements, plasmids, and phages. This indicates an ecosystem highly conducive to the horizontal transfer of resistance (Rahman et al., 2025). Similarly, surveillance in chicken farms has identified a core set of potentially mobile ARGs shared between birds and their environments. The gut microbiome composition correlates with the AMR profiles of *Escherichia coli* (Baker et al., 2023). The consequences of this agricultural AMR burden extend beyond farm boundaries. Environmental dissemination occurs through routes such as manure fertilization. This process not only enriches soil with ARGs but also facilitates their transfer into the gut microbiomes of exposed animals, as demonstrated in mouse models (Zhai et al., 2025). Furthermore, wild animals, including rats and passerine birds, can act as sentinels and bridges for AMR spread, with those inhabiting agricultural areas carrying a higher abundance and diversity of ARGs, including high-risk variants and zoonotic antimicrobial-resistant bacteria.

The genetic engine of this system is HGT. Within the densely populated colonic niche (10^11^–10^12^ CFU/g), conjugation, transduction, and transformation occur at frequencies several orders of magnitude higher than in oligotrophic environments (Borodovich et al., 2022). Recent network analyses have moved beyond simple detection to map plasmid conjugation networks and phage transduction networks. These are graph-theoretic models in which nodes represent bacterial species or strains, and edges represent documented HGT events (Borodovich et al., 2022). These networks reveal that certain bacterial taxa act as keystone hubs, facilitating ARG flow across otherwise unrelated phyla. The implication is profound, as the gut is not a mere bag of microbes but an organized, dynamic genetic exchange platform. Under the One Health framework, these dynamics create interconnected transmission pathways that we synthesize in this review.

**Figure 1:**
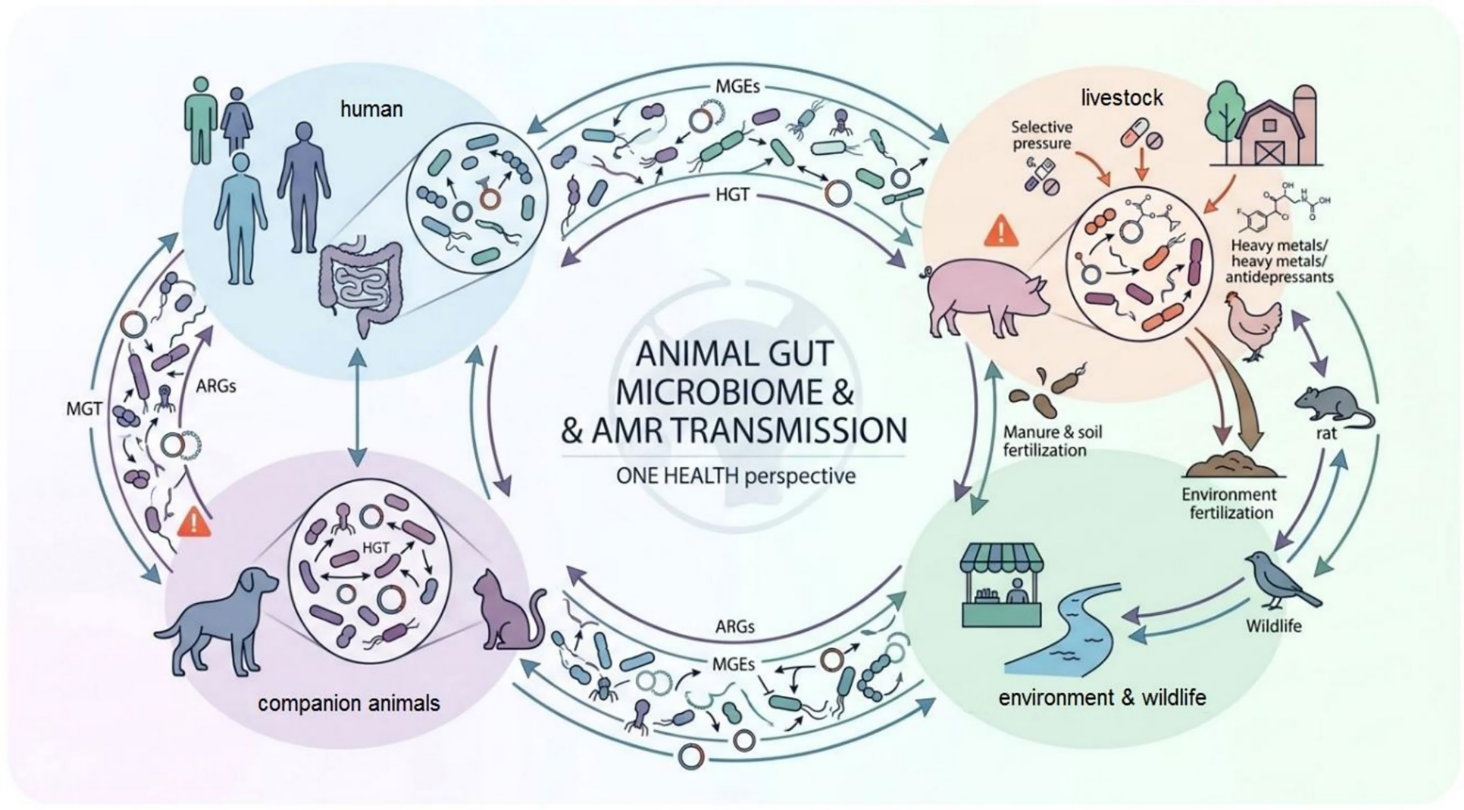
Conceptual model of the animal gut microbiome as a central hub for ARG transmission. The model shows three interconnected compartments (companion animals, livestock, humans) with bidirectional arrows indicating direct contact, foodborne, and environmental pathways. Microbial taxa are depicted as nodes; mobile genetic elements (plasmids, transposons, phages) shuttle ARGs across host species boundaries. Selection pressures (antibiotics, heavy metals) act on gut communities to amplify resistomes that disseminate through farm, household, and environmental interfaces.

The transmission dynamics of AMR are fundamentally governed by HGT mechanisms, including conjugation, transduction, and transformation, which allow ARGs to move between commensal bacteria, opportunistic pathogens, and true pathogens within the gut milieu (Borodovich et al., 2022). The human and animal gut microbiota is recognized as a significant reservoir for multidrug-resistant bacteria, such as extended-spectrum beta-lactamaseproducing *E. coli* (ESBL-EC). In dogs, carriage of ESBL-EC is associated with a distinct gut microbiome and resistome composition, characterized by an increased abundance of specific bacterial genera and ARGs like *cmlA* and *sul3* (Stege et al., 2023). Studies have identified robust associations between specific ARGs and specific transposons within the infant gut microbiota. Key players like *Klebsiella pneumoniae* and *E. coli* facilitate this synergistic horizontal transfer (Ding et al., 2024). This genetic mobility illustrates the gut as a crucible for the emergence of resistance. Even non-pathogenic environmental bacteria on plant foods can transfer resistance plasmids to clinical *E. coli* isolates. This allows these isolates to subsequently colonize the mammalian gut (Maeusli et al., 2020). The selection pressure is not limited to antibiotics. Other factors can also select for efflux pump mechanisms that confer crossresistance to antibiotics. These include exposure to heavy metals or even certain antidepressants. This further complicates the resistance landscape (Figure 1).

Under the “One Health” framework, which integrates human, animal, and environmental health, the role of the animal gut microbiome in AMR transmission is paramount. Zoonotic pathogens circulating between animal hosts and humans are of particular concern. For example, *Salmonella* Typhimurium transmission from poultry layer farms to nearby dairy herds has been documented, with strains accumulating additional resistance determinants, including to critically important extended-spectrum cephalosporins, during or after transmission events (Perry et al., 2024). Genomic surveillance of *E. coli* from swine and humans reveals shared high-risk zoonotic clones. It also highlights the pivotal role of horizontal gene transfer and plasmids in shaping distinct AMR profiles and enabling cross-species dissemination. Companion animals are increasingly recognized as reservoirs for critically important antimicrobial-resistant *E. coli*. Their strains show close genetic relatedness to human isolates, confirming pet-human transmission events (Sartori et al., 2026). The risks are amplified in settings with high human-animal interaction and poor hygiene, such as open markets, which can facilitate the transmission of resistant zoonotic pathogens (Hasan et al., 2025) (Figure 1). Therefore, we need a comprehensive understanding of three key areas: the ecological dynamics of the gut resistome in animal populations, the drivers of HGT, and the pathways of cross-boundary transmission. Such understanding is essential for designing effective, integrated interventions to mitigate the global AMR crisis (Vivarelli et al., 2026).

## Methods of literature search

This narrative review was conducted following standard guidelines for scoping and narrative reviews. A systematic literature search was performed in PubMed, Web of Science, and Scopus databases for articles published between January 2015 and December 2025. The search strategy combined keywords related to (i) gut microbiome, (ii) antimicrobial resistance, and (iii) animal hosts. Boolean operators (AND, OR) were used to combine search terms. Only peer-reviewed original research articles, reviews, and meta-analyses published in English were included. Conference abstracts, preprints, and non-English articles were excluded. Reference lists of included articles were manually screened for additional relevant studies. Priority was given to studies published in the last 5 years (2021–2025), with older landmark papers included for historical context.

### Animal gut microbiome

#### Composition and functional differences of gut microbiome in companion animals and livestock

The gut microbiome of companion animals, such as dogs and cats, exhibits a composition that shares notable similarities with the human gut microbiome, a relationship increasingly recognized under the “One Health” framework (Branck et al., 2024; Salamon et al., 2025; Lyu et al., 2025). This similarity is influenced by their close cohabitation with humans, which facilitates microbial exchange. It is further shaped by specific factors such as habitual diet, age, and medical.

interventions including antibiotic treatments (Shah et al., 2024; Lee D. et al., 2022; Banik et al., 2026). Diet exerts specific selective effects: high-protein/high-fat diets (typical of commercial pet foods) favor bile-tolerant taxa such as *Bacteroides* and *Clostridium*, while high-fiber diets promote saccharolytic bacteria like *Prevotella* and *Ruminococcus* that produce SCFAs. For instance, dietary interventions with various biotics are actively used to modulate the canine and feline gut microbiome (Wilson and Swanson, 2024). The microbial community in these animals is dominated by core phyla such as Firmicutes, Bacteroidetes, Proteobacteria, and Actinobacteria, but exhibits marked individual and interspecies variability (Banik et al., 2026). In contrast, livestock gut microbiomes are highly adapted to specific digestive physiologies. Ruminants (cattle, sheep) possess a forestomach (rumen) that supports a dense community of cellulolytic bacteria (Fibrobacter, Ruminococcus), methanogens, and protozoa, enabling breakdown of plant polysaccharides (Monteiro et al., 2022). Monogastric livestock (pigs) have a simpler stomach but a large hindgut fermentative compartment, with microbiomes shaped by consistent grain-based or high-fiber diets. Poultry possess a short gastrointestinal tract with ceca serving as primary fermentation sites, and their microbiomes are particularly sensitive to feed additives and environmental stressors. The statement that livestock microbiomes are “less diverse” requires qualification. Intensive, indoor, grain-fed systems typically exhibit reduced alpha diversity compared to extensive, pasture-based systems. This is due to uniform diet, limited environmental microbial input, and antibiotic exposure (Forcina et al., 2022). However, some intensive systems maintain diversity through continuous microbial seeding from feed and bedding (Forcina et al., 2022).

Critically for AMR, each host group exhibits distinct resistome characteristics. Ruminants frequently carry ARGs associated with tetracyclines and β-lactams, often linked to Bacteroidetes-associated mobile elements. Pigs are reservoirs for a wide range of ARGs including colistin resistance (*mcr-1*), often carried on plasmids with high conjugative potential. Poultry guts show enrichment of ESBL genes (*bla*_CTX-M_) and fluoroquinolone resistance determinants. Dogs and cats share ARGs with their owners, particularly aminoglycoside and tetracycline resistance genes, with household transmission being a dominant pathway (Table 1).

**Table 1:** Comparison of gut microbiome composition, dominant ARGs, and transmission risks across major animal groups.

Host group	Dominant phyla	Key functional features	Dominant ARG classes	Typical MGEs	Primary transmission route to humans	References
Ruminants (cattle)	Firmicutes, Bacteroidetes	Cellulose digestion, SCFA production	Tetracyclines, βlactams	Integrative conjugative elements	Foodborne (meat, dairy), environmental	Monteiro et al. (2022), Kim et al. (2023)
Pigs (monogastric)	Firmicutes, Bacteroidetes, Proteobacteria	Hindgut fermentation, high HGT capacity	Tetracyclines, macrolides, colistin (*mcr-1*)	Plasmids (IncX, IncI), transposons	Foodborne, direct contact (farm workers)	Rahman et al. (2025), Kim et al. (2023), Hickman et al. (2021)
Poultry	Firmicutes, Proteobacteria, Bacteroidetes	Cecal fermentation, rapid transit	ESBL (*bla*_CTX-M_), fluoroquinolones	Plasmids (IncF, IncI), phages	Foodborne (meat, eggs), environmental	Baker et al. (2023), Scicchitano et al. (2023), Ortega-Paredes et al. (2020)
Dogs/cats	Firmicutes, Bacteroidetes, Proteobacteria, Actinobacteria	Similar to human gut, dietresponsive	Aminoglycosides, tetracyclines, ESBL	Plasmids (IncF), transposons	Direct contact (petting, licking), household environment	Zhao et al. (2022), Cui et al. (2026), Branck et al. (2024), Jin et al. (2023)

#### The gut as a hotspot for horizontal gene transfer of resistance genes

The compositional and functional differences outlined above directly shape the capacity of the gut for horizontal gene transfer (Borodovich et al., 2022). A microbiome rich in Proteobacteria and harboring diverse mobile genetic elements creates a permissive environment for ARG exchange (Rahman et al., 2025). These compositional traits are more pronounced in livestock than in many companion animals (Rahman et al., 2025; Branck et al., 2024). The following section examines how the physical and ecological features of the gut turn this compositional predisposition into an active hotspot for genetic dissemination (McInnes et al., 2020; Vivarelli et al., 2026).

The animal gastrointestinal tract provides a uniquely conducive microenvironment for the HGT of antimicrobial resistance genes, functioning as a critical hotspot for their dissemination. This propensity is driven by several interconnected factors, including an exceptionally high density and diversity of bacterial populations, a continuous supply of nutrients, and a rich pool of MGEs such as plasmids, integrons, and transposons (Vivarelli et al., 2026). Within this dense ecological niche (10^11^–10^12^ CFU/g), conjugation, transformation, and transduction occur at frequencies several orders of magnitude higher than in oligotrophic environments (Borodovich et al., 2022). This enables the rapid movement of ARGs not only between different bacterial species but even across different genera (Du et al., 2022). This transfer can bridge commensal or environmental bacteria with opportunistic pathogens, effectively mobilizing resistance determinants into clinically relevant genetic backgrounds. Advanced metagenomic studies have begun to unravel the complex networks underpinning this exchange. For example, research reveals intricate “plasmid conjugation networks” and “bacteriophage transduction networks” within the gut. These are graphtheoretic models in which nodes represent bacterial species/strains and edges represent documented HGT events. They serve as molecular bridges facilitating cross-species AMR transmission (Scicchitano et al., 2023). These networks identify keystone taxa that act as hubs for ARG dissemination across otherwise unrelated phyla. For instance, a study on poultry farms has shown that clinically relevant ARGs and associated MGEs are shared among the gut microbiomes of poultry, farm workers, and their household members. The study also found that worker microbiomes act as a primary route for ARG dispersion from the farm system into human populations (Scicchitano et al., 2023). Similarly, comprehensive metagenomic analyses of mammalian gut microbiomes on the Tibetan Plateau identified numerous nonredundant ARGs and provided evidence for cross-species horizontal gene transfer events involving high-risk ARGs between human and non-human mammalian gut microbiota (Tian et al., 2025). The process is dynamic and influenced by various factors. The gut microbiome of saprophagous fauna is used for manure bioconversion. It can significantly attenuate the abundance and diversity of ARGs and associated MGEs from livestock waste. Network analyses implicate shifts in crucial bacterial phyla like Bacteroidetes and Firmicutes in this risk mitigation (Du et al., 2022). Furthermore, external pressures such as diet and co-infections can modulate these HGT networks. Dietary components in livestock feed can alter the gut microbial community, thereby influencing the resistome profile (Kogut, 2022). Concurrent infections, such as with gastrointestinal helminths in chickens, can modify the intestinal environment and microbiome composition and thereby create conditions that either favor or hinder the transfer and establishment of specific ARGs (Nemathaga et al., 2023). These insights collectively underscore that the gut is not merely a passive container for bacteria but an active arena where genetic material is constantly exchanged. The structure and dynamics of the resident microbial community play a decisive role in determining the scope and scale of AMR gene propagation across microbial and host species boundaries.

### Mechanisms for spread and maintenance of AMR in animal intestines

#### A unified selection framework: direct selection, co-selection, and cross-resistance

The persistence and spread of AMR in animal guts are governed by an interacting trio of selective mechanisms, namely direct selection, co-selection, and cross-resistance. Rather than acting in isolation, these forces operate simultaneously, creating a multi-dimensional selective landscape that sustains ARGs even in the absence of direct antibiotic pressure.

Direct selection is the most intuitive pathway, as antibiotic administration kills susceptible bacteria, enriching those carrying corresponding resistance genes. For instance, the ARMORD observational study demonstrated that recent exposure to specific antibiotics like piperacillin-tazobactam, meropenem, and clindamycin was associated with significant reductions in gut microbiome diversity and increased abundance of major ARGs (Peto et al., 2025). Similarly, a randomized trial on early-life antibiotics in infants showed that different empirical antibiotic combinations caused major shifts in gut microbial community composition and antimicrobial resistance gene profiles (Reyman et al., 2022). The magnitude of direct selection depends on drug class, dose, route, and duration, and is not uniform across individuals; baseline microbiome composition modulates the impact of the same antibiotic regimen.

Co-selection occurs when a non-antibiotic selective agent enriches ARGs because the resistance determinants are genetically linked. This operates through two mechanisms: (i) co-resistance, where different resistance genes reside on the same MGE; and (ii) crossresistance, where a single genetic mechanism confers resistance to multiple agents. Copper and zinc are common feed additives. They can co-select for antibiotic resistance. A mathematical modeling framework predicted minimum co-selective concentrations for metals, and its application to dairy farm slurry suggested the slurry was co-selective (Arya et al., 2021). However, experimental evidence is mixed. Some studies found that even high therapeutic doses of dietary copper did not cause significant co-selection of antibiotic resistance genes. This suggests that the impact depends on specific conditions and bacterial community context (Brinck et al., 2023; Forouzandeh et al., 2023). Beyond metals, environmental pollutants such as the fungicides carbendazim and chlorothalonil have been shown to elevate the abundance and diversity of ARGs. This may occur through stress on the microbiome and facilitation of HGT via MGEs (Song et al., 2022; Qiu et al., 2023).

Cross-resistance involves a single resistance mechanism providing protection against multiple structurally unrelated agents. The classic example is multidrug efflux pumps, which export tetracyclines, fluoroquinolones, β-lactams, as well as biocides and detergents. Fitness trade-offs govern their persistence. For instance, conditions that deplete the proton-motive force selected against pumps like AcrAB-TolC. Meanwhile, certain molecules could select for or against specific pumps. This illustrates the complex selection dynamics that can influence cross-resistance (Liu et al., 2024). This means that dietary components can inadvertently select for efflux pump-mediated resistance.

These three mechanisms are conceptually integrated rather than operating in separate silos. For example, an animal fed zinc-supplemented feed and subsequently treated with chlortetracycline will select for bacteria carrying a plasmid with both heavy metal and antibiotic resistance genes. Efflux pumps further broaden the resistant phenotype. The resistome is resilient, meaning it tends to persist even after selection is removed. This is explained by the stable integration of co-selected genes and MGEs into the gut microbial community’s genetic architecture, where they are often carried by core, abundant taxa that are not easily displaced (Peto et al., 2025; Jian et al., 2026). The overall stability and resilience of the microbiome significantly influence the persistence of AMR. Antibiotic perturbation can lead to failure of recovery and create new ecological niches that favor resistant bacteria (Mikolas et al., 2025).

#### The role of biofilm formation and persister cells

**Figure 2:**
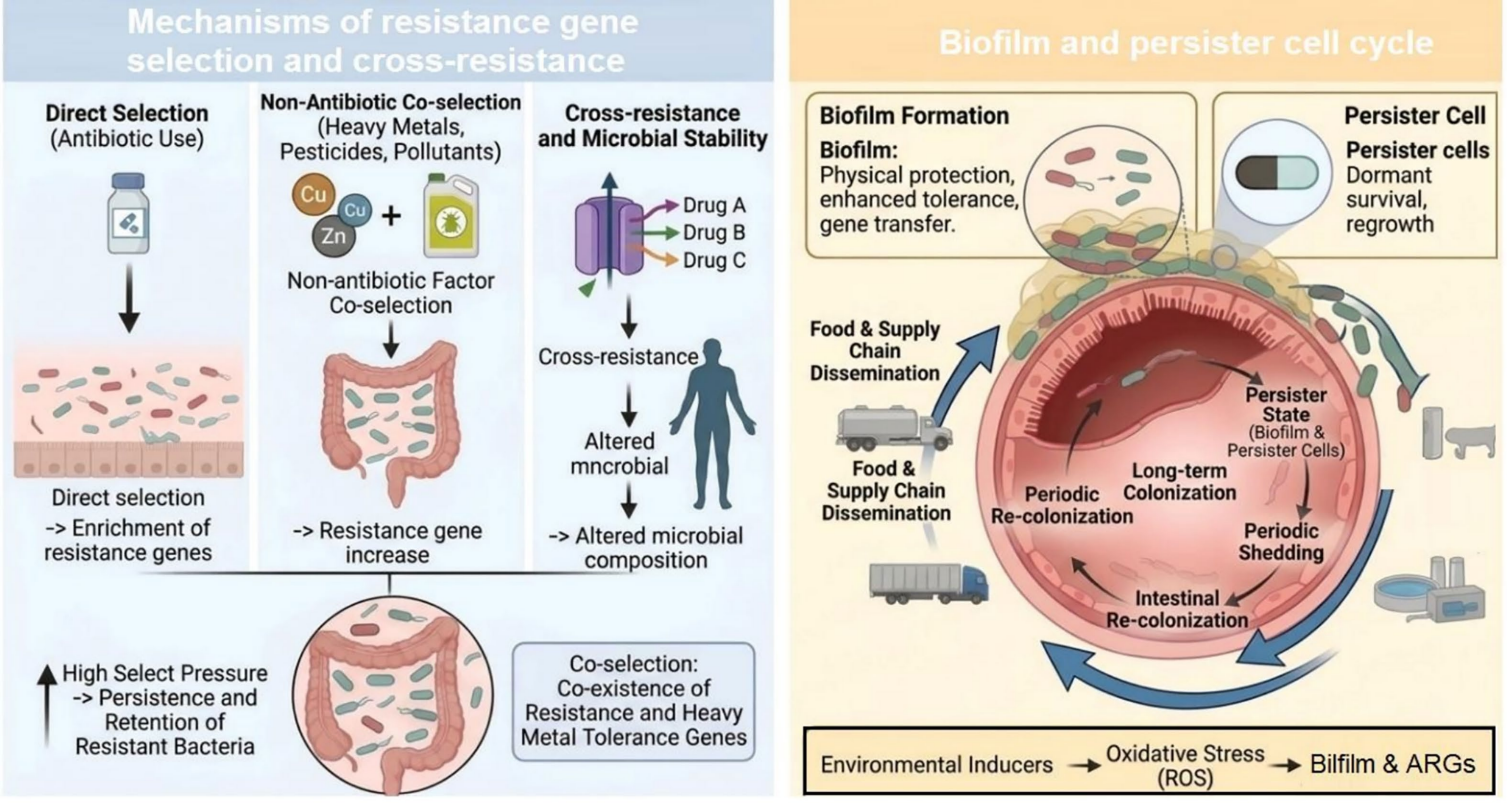
Mechanistic flow diagram of AMR selection and persistence in the animal gut. The figure is organized into three interconnected modules: (1) direct selection by antibiotics, shown as a filter that enriches ARG-carrying bacteria; (2) co-selection and cross-resistance, depicted as overlapping selective pressures from heavy metals, disinfectants, and biocides acting on shared efflux pumps and MGEs; and (3) the biofilm-persister cycle, illustrating how biofilm formation on mucosal surfaces creates a protected niche where bacteria survive antibiotic exposure, with persister cells serving as a reservoir for later regrowth and dissemination. Arrows connect these modules to the gut lumen and to environmental shedding, emphasizing the cyclical nature of AMR maintenance.

Within the intestine, bacteria, particularly certain members of the Enterobacteriaceae, can form biofilms on mucosal surfaces or particles (Figure 2). These biofilms provide physical protection, significantly enhancing bacterial tolerance to antibiotics, and offer a stable platform for genetic exchange, including the transfer of resistance genes. Biofilm formation is a recognized challenge in the dairy industry. Bacteria such as *Staphylococcus aureus*, *Bacillus* spp., *Listeria monocytogenes*, and *Pseudomonas* spp. can persist on equipment, leading to product spoilage and food safety issues. These biofilm bacteria also demonstrate increased resistance to biocides and antibiotics compared to their planktonic forms (Goetz et al., 2025). Persister cells represent a bacterial subpopulation that is non-genetically resistant to antibiotics. They can survive antibiotic treatment and resume proliferation once the treatment ceases, acting as seeds for antimicrobial resistance recurrence and transmission. The dynamics of bacterial invasion and fitness in complex communities, such as the gut microbiota, are critical for understanding how resistant strains like *E. coli* can establish and persist. A mechanistic framework has been developed to quantify fitness differences between co-invading strains in a multispecies context. This framework underscores how the frequencies of resident species can modulate selection coefficients, which is relevant for predicting the invasion success of antibiotic-resistant pathobionts (Ferreira Amaro Freire et al., 2025). Biofilms and persister cells work together. They promote long-term colonization and periodic shedding of resistant strains. This increases the risk of environmental transmission. This is particularly concerning for pathogens that can survive in the environment for extended periods. For example, *Streptococcus suis*, a pathogen associated with livestock, can persist in the environment and carry antibiotic resistance genes. Induction experiments showed that sub-inhibitory concentrations of the disinfectant peracetic acid could increase *S. suis* resistance to the antibiotic tiamulin. This triggers emergency responses such as increased reactive oxygen species and biofilm formation. These responses illustrate a co-selection mechanism that could enhance persistence and transmission (Ma et al., 2025).

The persistence of resistant bacteria is also influenced by cleaning and disinfection practices. A recent study on livestock transport trailers demonstrated that standard cleaning and disinfection protocols significantly reduced but did not eliminate drugresistant *E. coli*, indicating that vehicles can act as fomites for AMR spread if cleaning is inadequate (Urbaniec et al., 2025). Furthermore, the in-hospital acquisition of resistant bacteria in veterinary settings highlights this risk. A study in an Italian Veterinary Teaching Hospital found a high frequency of carbapenem-resistant Enterobacterales (CRE) acquisition in dogs and cats during hospitalization, linked to factors like length of stay and treatment with piperacillin—tazobactam. This suggests potential cross-selection and highlights companion animals as a reservoir for resistant organisms with One Health implications (Scarpellini et al., 2025). The interplay between biofilm, persisters, and environmental contamination creates a cycle that sustains AMR. In wastewater treatment, which is a known hotspot for AMR, biofilms can protect bacteria and facilitate gene exchange. Studies on the removal of ARGs in wastewater treatment plants have shown that biological treatment reduces but does not completely eliminate ARGs, and biofilms in systems like oxidation ditches may contribute to their persistence (Li Z. et al., 2024). Advanced treatment processes like electrochemical oxidation and electro—Fenton have been explored for inactivating antibiotic-resistant bacteria and degrading intracellular and extracellular ARGs. These approaches address the challenge posed by persistent cells and genetic material (Chen et al., 2020).

A critical synthesis of current evidence reveals that despite the wealth of correlative data, several unresolved controversies and causal gaps remain. First, the relative contribution of *in vivo* HGT versus clonal expansion of resistant strains to the overall gut resistome load remains unknown. Most metagenomic studies cannot distinguish between active transfer and passive enrichment (Borodovich et al., 2022; Zhao et al., 2022; Cui et al., 2026). Second, while co-selection by heavy metals is mechanistically plausible, the minimum selective concentrations under realworld animal husbandry conditions have not been experimentally validated for most metal-ARG pairs (Arya et al., 2021; Brinck et al., 2023). Third, biofilm-associated persistence is well documented *in vitro*, but direct *in vivo* evidence linking biofilm formation to AMR transmission outcomes in the animal gut is scarce (Goetz et al., 2025; Urbaniec et al., 2025; Scarpellini et al., 2025). Fourth, the causal direction between microbiome perturbation and ARG acquisition remains ambiguous. It is unclear whether antibioticinduced dysbiosis precedes and enables ARG invasion, or whether ARG carriage itself drives community shifts (Zhao et al., 2022; Cui et al., 2026; Peto et al., 2025; Reyman et al., 2022). Future research should prioritize controlled longitudinal experiments and metagenomic approaches that track plasmid and transposon dynamics at single-cell resolution.

### Main factors driving the spread of AMR

#### Antimicrobial use practices in animal production and healthcare

In livestock production, antimicrobials are extensively employed for therapeutic, prophylactic, and growth-promoting purposes. Such sub-therapeutic, long-term exposure constitutes the strongest selective pressure driving the generation and dissemination of antimicrobial resistance within gut microbiomes (Enshaie et al., 2025). The global estimate of veterinary antibiotic consumption ranges from 63,000 to 106,000 tons annually, with tetracyclines, sulfonamides, β-lactams, and macrolides being predominant classes (Abate and Birhanu, 2025). Projections indicate that under a business-as-usual scenario, global livestock antibiotic use could reach approximately 143,481 tons by 2040, a significant increase from 2019 levels (Acosta et al., 2025). The use of antimicrobials in livestock, particularly for growth promotion and metaphylaxis, exacerbates selective pressures, fostering the proliferation of multidrugresistant bacterial strains such as *E. coli*, *Salmonella* spp., and *Staphylococcus aureus* (Matheou et al., 2025). This practice is a major concern in developing countries, where antimicrobial use in animal husbandry is common and often unregulated, leading to environmental contamination with antibiotic residues and resistance genes (Abate and Birhanu, 2025). While many countries have implemented bans or restrictions on antimicrobial growth promoters, the legacy of the antibiotic era persists, shaping preventive efforts and reinforcing data-driven control in livestock management (Hinchliffe, 2025).

In companion animal clinical practice, antimicrobials are prescribed for various infections, but inappropriate prescribing similarly perturbs the intestinal microbiome and selects for resistant strains. Examples include overuse of broad-spectrum antibiotics and inadequate treatment duration (Taylor et al., 2022). Surveys from multiple countries, including the United States, Chile, Hong Kong, and Japan, consistently report common empirical prescribing of antimicrobials for conditions such as pyoderma, diarrhea, and urinary tract infections. This prescribing often occurs without prior culture and susceptibility testing.

Contributing factors include financial burden on owners, treatment delays, and time constraints (Karalliu et al., 2025; Galarce et al., 2021; Makita et al., 2021). Studies indicate that awareness of antimicrobial use guidelines among veterinarians is associated with lower prescribing levels for certain conditions, highlighting the importance of stewardship programs (Taylor et al., 2022). The route of administration significantly influences the impact on the gut microbiota. For instance, systemic antimicrobials are often prescribed empirically and have a broad effect. In contrast, topical antimicrobials have a more localized impact, and their use can be quantified and potentially reduced through stewardship programs (Kardomatea et al., 2023). Different antimicrobial classes and administration routes exert distinct selective pressures on the gut microbial community, thereby shaping unique profiles of antibiotic resistance genes. For example, the use of critically important antimicrobials (CIAs), such as third- and fourth-generation cephalosporins and fluoroquinolones, is of particular concern due to their importance in human medicine (Bonzelett et al., 2022) (Figure 2). Monitoring efforts reveal challenges in accurately quantifying companion animal antimicrobial use, especially for parenteral products. This suggests that actual consumption may be significantly higher than estimated (Glavind et al., 2022). This underscores that antimicrobial use practices in both livestock and companion animals are fundamental drivers of AMR selection within animal gut microbiomes. These practices are driven by clinical need, production demands, and various socio-economic factors, and they have direct implications for resistance gene pools that can be shared across the One Health continuum (Figure 2).

#### Husbandry management and environmental factors

Intensive, high-density farming systems significantly increase close contact among animals and opportunities for fecal-oral transmission, greatly accelerating the spread of resistant bacteria and genes within populations (Tanquilut et al., 2020). Quantitative assessments of biosecurity highlight that low scores in areas such as live animal transport and infrastructure can increase disease incursion risks. This may consequently increase antimicrobial use and resistance spread (Tanquilut et al., 2020). In such settings, the sharing of antimicrobial resistance genes between animals and humans via horizontal gene transfer is extensive. Studies in livestock systems show higher AMR prevalence in medium-scale farms and clear zoonotic transmission gradients, for example, colistin resistance from pigs to handlers and their contacts (Silvestro et al., 2025). Feed composition, stress events, and hygiene conditions are key factors that alter the gut environment. They influence microbial community structure and the frequency of HGT, thereby indirectly driving AMR evolution. For example, dietary components and stressors can disrupt the gut-microbiota-brain axis, leading to dysbiosis and systemic inflammation, which may further influence resistance dynamics (Gu et al., 2020).

**Figure 3:**
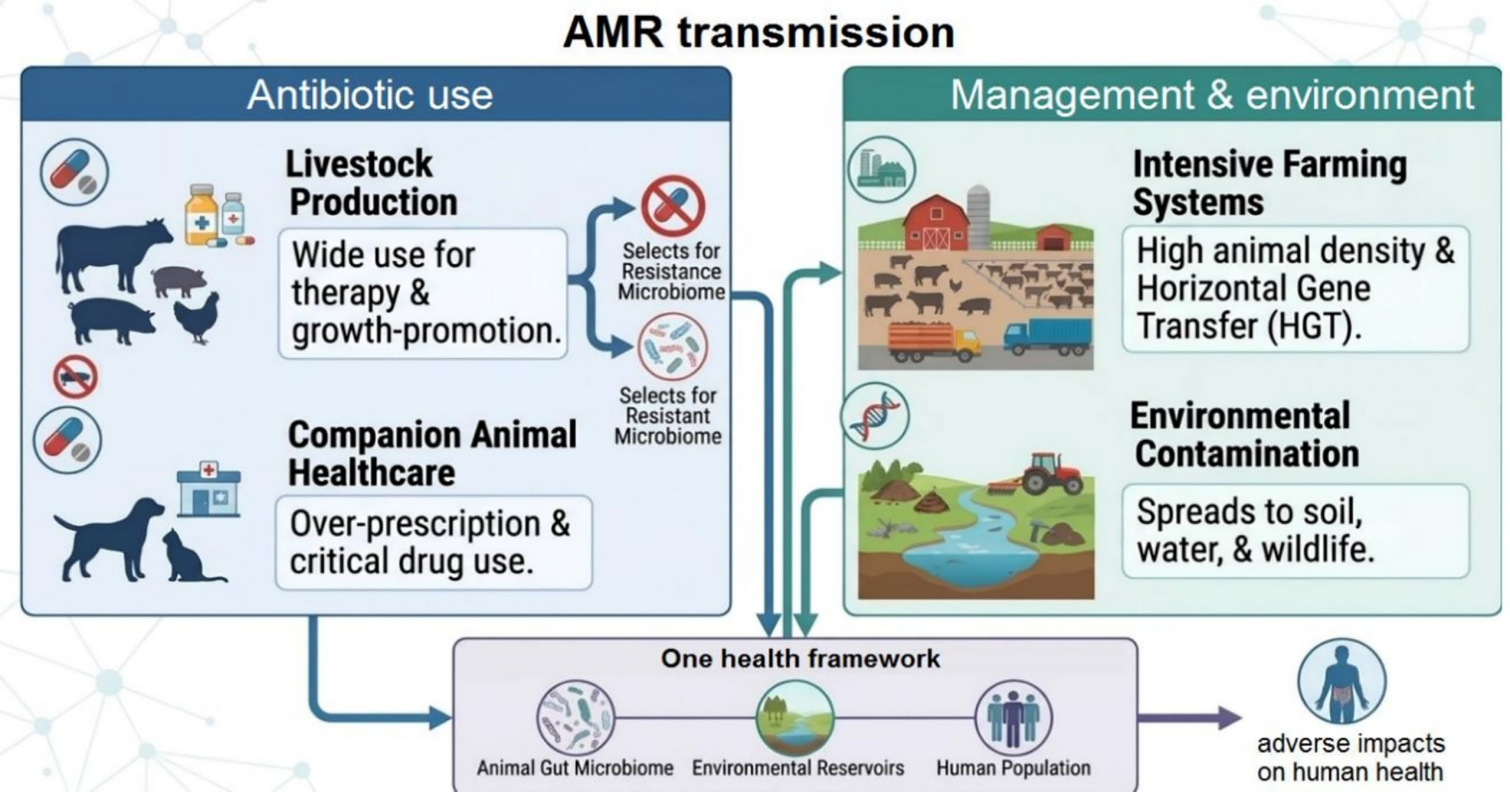
Drivers and transmission pathways of AMR under the One Health framework. The diagram is structured as a series of nested circles. The innermost circle represents the animal gut microbiome where ARGs are selected and amplified. The middle circle depicts on-farm drivers. The outermost circle encompasses off-farm pathways. Colored arrows indicate the flow of resistant bacteria and ARGs from livestock and companion animals to environmental compartments (soil, water, air) and ultimately to human exposure routes. Quantitative indicators link qualitative pathways to measurable surveillance metrics.

Farm environments, including soil, water, and equipment, become contaminated with resistant bacteria and ARGs from animal manure, creating persistent reservoirs and cycles of resistance. Livestock waste from family—operated farms is a significant and often overlooked source of ARGs and other contaminants, which pose risks to surrounding farmland soils upon application (Li et al., 2023; Zhang et al., 2020). Manure composting can reduce the relative abundance of ARGs and mobile genetic elements. However, residues may still pose risks after application, and the dissipation of ARGs is linked to factors such as moisture, bacterial succession, and the profile of biocide and metal resistance genes (Lee S. et al., 2022). These contaminated farm environments can act as sources for the diffusion of resistance into the wider environment and to wildlife. Research indicates that wildlife, such as feral swine and coyotes, can harbor more abundant ARGs compared to grazing cattle, suggesting potential transmission at the wildlife-livestock interface and that wildlife could be a source of AMR colonization in livestock (Sun et al., 2025). The environmental fate of ARGs from livestock farming involves dissemination through pathways like manure application, wastewater discharge, and aerosol diffusion, eventually reaching humans and accumulating in the intestinal microbiota, impacting human health (Li T. et al., 2024). Factors influencing this spread include the interplay of microbial communities, antibiotics, heavy metals, and other environmental parameters (Li T. et al., 2024). Therefore, husbandry practices and environmental factors are critical amplifiers of AMR, facilitating the maintenance and spread of resistance determinants within animal populations and creating bridges for transmission to wildlife, the environment, and ultimately, human populations under the One Health framework (Figure 3).

### Cross-species transmission pathways and the “One Health” interface

#### Direct and indirect transmission pathways from animals to humans

The environmental reservoirs and farm-level drivers discussed above do not remain confined to animal production settings. Rather, they seed resistant bacteria and ARGs into surrounding soil, water, and wildlife, creating pathways that extend beyond the farm gate. The next section synthesizes these direct contact, foodborne, and environmental pathways into a coherent One Health transmission model. It then analyzes the surveillance and intervention challenges that arise when trying to disrupt such interconnected routes. The food chain represents a critical indirect transmission route. Specifically, consumption of undercooked or contaminated meat from food-producing animals is a primary source of foodborne antimicrobial-resistant bacterial infections in humans (Fastl et al., 2023). Intensive farming practices often involve routine antibiotic use. These practices foster antimicrobial-resistant bacteria in animal gastrointestinal tracts (Vivarelli et al., 2026). The bacteria and their ARGs are excreted and can contaminate meat products during slaughter and processing, leading to cross-contamination (Ortega-Paredes et al., 2020). For instance, studies on broiler farms and carcasses in Ecuador identified them as important reservoirs of multi-drug resistant *E. coli* carrying ESBL (*bla*_CTX-M_) and colistin resistance (*mcr-1*) genes, directly linking poultry production to human exposure (Ortega-Paredes et al., 2020). Similarly, retail meat serves as a reservoir for multidrug-resistant bacteria like *Acinetobacter baumannii*, with genomic features mirroring clinical strains, highlighting the risk of zoonotic transmission via the food chain (Vanishree et al., 2026). Animal manure used as fertilizer and wastewater from farms introduces vast quantities of antimicrobial-resistant bacteria and ARGs into soil and water systems (Zhai et al., 2025). Early-life exposure of mice to manure-fertilized soil was shown to profoundly shape their gut antibiotic resistome, significantly increasing the abundance of tetracycline resistance genes like *tet(Q)* (Zhai et al., 2025). This environmental contamination can subsequently pollute crops and drinking water sources, establishing an environment-to-human transmission loop (Samtiya et al., 2022). Research confirms that ARGs can be transferred from environmental bacteria on plants to human gut bacteria. This is demonstrated by the in planta transfer of resistance plasmids from *Acinetobacter baylyi* to *E. coli*, which subsequently colonized the mouse gut (Maeusli et al., 2020). Collectively, these pathways illustrate a complex web where resistant determinants circulate freely between human, animal, and environmental compartments, driven by agricultural practices, close human-animal bonds, and inadequate waste management.

While the pathways described above are qualitatively well established, quantitative estimates of their relative contributions remain limited. Meta-analyses of foodborne outbreaks attribute approximately 20–30% of human infections with extended-spectrum β-lactamase (ESBL)-producing *E. coli* to livestock-associated strains, although source attribution varies widely by region and production system (Ludden et al., 2019). Companion animals account for an estimated 5–15% of household ARG carriage in pet-owning households, based on metagenomic abundance comparisons (Cui et al., 2026). Environmental transmission via manure-fertilized soil contributes a lower relative proportion of human ARG acquisition, estimated at less than 5% in high-income countries. However, it may be more significant in low-income settings, which are characterized by direct human–livestock contact and untreated wastewater use (Zhai et al., 2025). Quantitatively, ARG abundance per 16S rRNA gene or gram of feces in livestock guts is typically 1–2 orders of magnitude higher than in companion animals or humans, reflecting continuous selection pressure (Enshaie et al., 2025). However, absolute risk estimates, such as the probability of ARG transfer from an animal to a human given a single exposure event, remain unknown. Furthermore, no studies have established dose–response relationships or minimum infectious doses for ARG-carrying commensals. Future quantitative microbiological risk assessment (QMRA) modeling, combined with longitudinal cohort studies and whole-genome sequencing, is urgently needed to prioritize intervention targets.

#### Challenges for “One Health” surveillance and intervention

Implementing effective “One Health” strategies to combat antimicrobial resistance faces substantial challenges in both surveillance and intervention, stemming from systemic fragmentation and complex socio-economic trade-offs. A core surveillance challenge is the need to establish integrated, standardized AMR monitoring systems that seamlessly combine data from human, animal, and environmental samples (Fitzpatrick et al., 2021). Current data remain largely fragmented, hindering the ability to track the flow of resistant bacteria and ARGs across the One Health interfaces (Bulteel et al., 2021). Advanced technologies like whole-genome sequencing (WGS) are crucial for high-resolution tracing of transmission trajectories. For example, genomic surveillance of *Salmonella enterica* serovar Dublin in the U. S. revealed a highly related population across bovine, human, and environmental sources, yet also identified source-dependent differences in ARG profiles, emphasizing the need for integrated genomic analysis to understand transmission dynamics (Kenney et al., 2025). Similarly, a study in India found limited evidence of spillover of antimicrobial-resistant *K. pneumoniae* clones from animal/environmental reservoirs to humans in a specific setting, challenging assumptions and highlighting the importance of context-specific, integrated surveillance to accurately map resistance flows (Jacob et al., 2024). The intervention landscape is equally fraught with difficulties. Restricting antimicrobial use in animals is a cornerstone measure but requires balancing animal welfare, productivity, and economic pressures (Loayza-Villa et al., 2021). A study on swine demonstrated that removing prophylactic antimicrobials over two generations did not negatively affect weight gain but was also insufficient to reduce the richness and abundance of ARGs in the gut resistome, indicating the persistence of resistance once established and the complexity of intervention (Loayza-Villa et al., 2021). Coordinating policies across agriculture, public health, and environmental sectors is notoriously difficult (Al-Khalaifah et al., 2025). Global initiatives like the WHO Global Action Plan underscore the need for international collaboration, but implementation varies widely, especially in low- and middle-income countries (LMICs) where surveillance systems may be weaker (Malik et al., 2023). Furthermore, a significant barrier is the limited awareness and understanding of AMR among the public, farmers, and even healthcare professionals, which directly impacts the adoption of stewardship practices and intervention efficacy (Chambers et al., 2020). A rapid review highlighted the urgent need for psychological and behavioral research to identify determinants of antibiotic use and to evaluate theory-based interventions aimed at reducing use in food production animals (Chambers et al., 2020). Economic and governance factors also play a critical role. An ecological study across 30 countries found that factors such as per capita health expenditure and governance quality were significantly associated with AMR rates. This suggests that addressing AMR requires more than just reducing antibiotic consumption; it must also include broader socio-economic development and strengthening of healthcare systems (Silva et al., 2021). Ultimately, overcoming these challenges demands a sustained, multidisciplinary effort that integrates robust, cross-sectoral surveillance with context sensitive interventions. This effort must be supported by global cooperation and increased investment in public and professional education.

Current One Health AMR surveillance is hampered by significant limitations and knowledge gaps, remaining largely siloed with human, animal, and environmental data rarely integrated at the individual or farm level (Fitzpatrick et al., 2021; Bulteel et al., 2021). Most studies rely on cross-sectional sampling, which captures only a snapshot and cannot establish transmission directionality or timing. Furthermore, the lack of standardized protocols for sample processing, sequencing, and bioinformatic analysis across sectors precludes meaningful meta-analysis and global comparisons (Al-Khalaifah et al., 2025; Malik et al., 2023). The attribution of ARG transmission to specific pathways is almost never quantified with source-tracking models that account for population-level exposure. A critical unmet need is the development of causal inference frameworks, such as Bayesian network models combined with longitudinal whole-genome sequencing. These frameworks would help distinguish genuine cross-species transmission from shared environmental acquisition or convergent evolution (Vivarelli et al., 2026; Hickman et al., 2021).

Recent advances in molecular and computational tools offer transformative potential to address the limitations of current AMR surveillance. Long-read sequencing platforms such as Oxford Nanopore and PacBio enable complete assembly of plasmids and the chromosomal contexts of ARGs, allowing unambiguous tracking of MGE-mediated transmission without the fragmentation inherent to short-read metagenomics (Li Z. et al., 2024; Sun et al., 2025). Metatranscriptomics can differentiate actively expressed ARGs from passively carried DNA, providing functional rather than merely compositional resistome data (Li Z. et al., 2024). Singlecell techniques, including droplet-based microfluidics coupled with fluorescenceactivated cell sorting, allow direct observation of HGT events in complex microbial communities, moving beyond correlation to causation (Borodovich et al., 2022; Scicchitano et al., 2023). For rapid point-of-care or on-farm diagnostics, CRISPR-based platforms and isothermal methods enable sensitive, specific, low-cost detection of high-risk ARGs such as *bla*_NDM_, *mcr-1*, and *tet(X)* within hours (Vivarelli et al., 2026; Jin et al., 2023). Machine learning and network inference algorithms applied to large-scale metagenomic datasets can predict plasmid host ranges and identify previously unrecognized ARG-MGE associations (Scicchitano et al., 2023; Baker et al., 2023). These technologies are not yet widely deployed in routine One Health surveillance. Their integration into national monitoring programs represents a high-priority research and investment area, particularly in low- and middle-income countries where laboratory infrastructure is limited (Hickman et al., 2021; Al-Khalaifah et al., 2025).

### Microbiome-based management strategies for AMR mitigation

#### Probiotics and competitive exclusion strategies

Probiotics, such as *Lactobacillus* and *Bifidobacterium* strains, function as effective natural alternatives to antibiotics by employing multiple mechanisms to inhibit the colonization and proliferation of potential pathogens and antimicrobial-resistant bacteria in the gastrointestinal tracts of companion and food-producing animals. A primary mechanism is competitive exclusion. Beneficial bacteria physically occupy adhesion sites on the intestinal epithelium, thereby preventing pathogens from establishing a foothold. For instance, specific strains like *Lactobacillus casei* ATTC334 and *Bifidobacterium breve* JCM1192 exhibit strong adherence to Caco-2 intestinal epithelial cells, which directly reduces the recovery of *Salmonella* Typhimurium from cecal tonsils in broiler chickens through this competitive binding (El-Sharkawy et al., 2020). Similarly, lactic acid bacteria (LAB) isolated from poultry feces, such as *Lactobacillus salivarius* and *Lactobacillus johnsonii*, demonstrate significant ability to hamper the adhesion of *Salmonella* Typhimurium to Caco-2 cells in exclusion, competition, and displacement assays (Salehizadeh et al., 2020). Beyond simple competition for space, probiotics produce a range of antimicrobial substances. These include bacteriocins, organic acids, short-chain fatty acids (SCFAs), and hydrogen peroxide, which create a hostile microenvironment for pathogens (Kart et al., 2026). The production of organic acids, particularly lactic acid, lowers the intestinal pH, further inhibiting acidsensitive pathogens. Certain strains also enhance the host’s mucosal barrier integrity and modulate immune responses, contributing to a more resilient gut ecosystem. For example, *Bifidobacterium infantis* BL2416 has poor epithelial cell binding but still reduced *Salmonella* Typhimurium recovery and increased cytokine production in broilers, suggesting an immunomodulatory role (El-Sharkawy et al., 2020). The yeast probiotic *Saccharomyces boulardii* also operates through a conjunction of pathways including competitive exclusion, production of antimicrobial peptides, and immune modulation (Pais et al., 2020). By improving gut health and directly antagonizing pathogens, probiotics reduce the incidence of infectious diseases, thereby diminishing the need for therapeutic antimicrobials and lowering the selective pressure that drives AMR. This positions them as crucial tools for antibiotic stewardship. Research highlights their role in improving animal productivity, such as body weight gain and feed conversion rate in *Salmonella*infected broilers, while simultaneously mitigating AMR risks (El-Sharkawy et al., 2020; Naeem and Bourassa, 2025).

Future directions focus on developing next-generation probiotics or defined consortia with enhanced or specific functionalities. This includes strains engineered or selected for superior competitive exclusion, targeted antimicrobial production, or the ability to inhibit HGT of resistance determinants (McInnes et al., 2020; Borodovich et al., 2022). Advanced delivery systems, such as encapsulation in biopolymers or synthetic carriers, are being optimized to improve probiotic viability under gastrointestinal stressors and ensure controlled release. This enhances their therapeutic consistency against multidrug-resistant bacteria (Kart et al., 2026). The concept applies not only to dietary supplements but also to environmental sanitation. Formulations such as probiotic-infused activated charcoal/hydroxyapatite microbeads establish protective biofilms on high-risk surfaces, offering rapid disinfection and longterm competitive exclusion of antibiotic-resistant pathogens (Alqumber, 2026). Combining probiotics with other biocontrol agents, such as bacteriophages, presents a novel synergistic strategy informed by the understanding of the gut resistome as a transmission hub (Vivarelli et al., 2026; Du et al., 2022). *In vitro* studies demonstrate that co-administration of phages with antimicrobial-susceptible commensal *E. coli* can effectively decolonize extended-spectrum cephalosporin-resistant *E. coli* by coupling phage-mediated lysis with the prevention of phage-resistant mutant regrowth through competition (Laird et al., 2022). This multi-pronged approach underscores the potential of refined probiotic applications for precise microbiome modulation to combat AMR within a One Health framework.

Despite this promise, several critical limitations temper the direct translation of probiotic research into practice. Strain-specificity is extreme, as a *Lactobacillus* strain effective in one chicken breed may fail in another due to differences in mucus glycan composition or immune development. Host dependency is profound, results from *in vitro* assays often fail to predict outcomes in pigs, cattle, or companion animals due to differences in gut transit time, pH, and resident microbiota (Kart et al., 2026). Reproducibility issues abound: meta-analyses of probiotic trials in livestock show significant heterogeneity, largely unexplained by study design. Regulatory and scalability challenges exist for defined consortia and next-generation probiotics, as they require lengthy and expensive safety approval. Moreover, in many jurisdictions, probiotics for animals are regulated as feed additives, which allows poorly characterized products to reach market (Naeem and Bourassa, 2025). Finally, ecological resistance must be considered. The resident gut microbiota can exclude introduced probiotics via priority effects. Future research should prioritize strain selection using host-specific *in vitro* gut models and develop defined consortia rather than single strains.

#### Prebiotics, postbiotics, and fecal microbiota transplantation

Prebiotics, postbiotics, and fecal microbiota transplantation (FMT) represent complementary and increasingly sophisticated strategies for modulating the gut ecosystem to suppress antimicrobial-resistant bacteria in animals. Prebiotics such as fructooligosaccharides (FOS), galactooligosaccharides (GOS), isomalto-oligosaccharides (IMO), and inulin, are non-digestible food ingredients that selectively stimulate the growth and/or activity of beneficial indigenous bacteria like *Bifidobacterium* and *Lactobacillus* (Yue et al., 2025). By promoting a healthier and more stable microbial community structure, prebiotics enhance the gut’s ecological resistance to invasion by pathogens and resistant bacteria. This indirect suppression occurs through mechanisms such as the production of SCFAs by fermentative bacteria. These SCFAs lower luminal pH and exhibit direct inhibitory effects on pathogens. At the same time, they strengthen the intestinal barrier and modulate host immunity. The resultant optimized microbiota improves nutrient utilization, overall gut health, and animal performance, thereby reducing the incidence of diseases that would otherwise necessitate antibiotic use (Yue et al., 2025; Atuahene et al., 2025). Postbiotics, defined as inanimate microorganisms and/or their components/metabolites that confer a health benefit, offer a promising alternative to live probiotics. These include heat-inactivated bacterial cells, cell-free supernatants, and purified metabolites like bacteriocins, SCFAs, and peptides. Their advantages include high safety profiles, greater stability during storage and processing, and consistent biological activity. Postbiotics exert health-promoting effects through immunomodulation, enhancement of epithelial barrier function, and direct antimicrobial activity. A study in an *in vitro* canine gut model used a postbiotic derived from heat-inactivated *Lactobacillus helveticus* HA-122. When administered during and after antibiotic treatment, this postbiotic effectively mitigated dysbiosis. It accelerated the recovery of total bacterial load and diversity, preserved SCFA concentrations, and reduced the bloom of Enterobacteriaceae, showcasing its potential to restore gut homeostasis after antibiotic disturbance (Deschamps et al., 2025). Similarly, metabolites from beneficial microbes can inhibit pathogen adhesion and biofilm formation. The use of postbiotics is particularly attractive in settings where the administration of live microbes is challenging or regulated.

Fecal microbiota transplantation (FMT), the transfer of processed fecal material from a healthy donor to a recipient, aims to rapidly reconstruct a healthy, diverse, and functional gut microbiota. In veterinary medicine, FMT has been explored to treat dysbiosis-associated conditions and shows potential for decolonizing gut reservoirs of AMR determinants. For instance, early-life interventions like FMT or rumen microbiota transplantation in calves can effectively alleviate diarrhea and promote a healthy gut microbiota development, offering an antibiotic alternative (Du et al., 2023). The principle is to introduce a complete, resilient microbial community that can outcompete and displace established resistant bacteria. However, the application of FMT in animals faces significant hurdles that require deeper research. Key challenges include the standardization of preparation protocols and ensuring the safety and long-term stability of the transplanted microbiota. Another major risk is the inadvertent transfer of pathogens or undesirable genetic elements, including antimicrobial resistance genes themselves. Furthermore, the ecological dynamics governing engraftment success and the specific mechanisms by which a transplanted microbiota eliminates resistant bacteria are not fully understood. Future research must address these gaps to develop safe, effective, and standardized FMT protocols for use in livestock and companion animals. This would position FMT as a viable tool within integrated AMR mitigation strategies under the One Health paradigm.

However, beyond the hurdles already noted, FMT faces additional challenges. Standardization of donor screening, preparation methods (fresh, frozen, lyophilized), and administration routes varies widely, precluding meta-analysis. Safety risks include the transmission of antibiotic-resistant organisms themselves, as ARGs can be present in healthy donor feces. Rigorous screening for high-risk ARGs is essential but resourceintensive. Engraftment dynamics are poorly understood, as the conditions required for a transplanted microbiota to permanently displace a resident resistant community remain unclear (Du et al., 2023). Regulatory status of veterinary FMT is ambiguous, classified as a drug, biologic, or feed additive depending on jurisdiction, creating uncertainty for clinical adoption. Priority research areas include double-blinded, placebo-controlled FMT trials in livestock with AMR colonization and the development of defined synthetic microbial consortia that mimic the anti-AMR effects of FMT without the risks.

## Conclusion

In this review, we clarified three previously underappreciated realities about the role of the animal gut microbiome in AMR transmission. First, the gut functions as an active network hub where horizontal gene transfer rates far exceed those in environmental compartments. Second, co-selection from heavy metals, disinfectants, and nonantimicrobial agents can sustain resistance even without direct antibiotic use. Third, intervention success is highly context-dependent. Among currently identified strategies, phage-probiotic synergy showed the highest promise for decolonizing resistant strains, postbiotics offered a safer and more stable alternative to live probiotics, and antibiotic stewardship combined with quantitative biosecurity measurably reduces AMR prevalence. Fecal microbiota transplantation and copper-based feed alternatives remain promising but uncertain, lacking rigorous controlled trials and showing context-dependent or inconsistent effects. Immediate priorities include integrating non-antibiotic selective agents into surveillance, developing host-specific validated probiotic consortia, regulating mobile genetic elements as reportable targets, and establishing open-access HGT network databases.

Looking forward, translational implementation requires embedding these findings into veterinary and public health practice. Examples include on-farm metagenomic monitoring of resistome dynamics and rapid CRISPR-based diagnostics for high-risk ARGs. Policy implications are substantial, as current regulations focus on antibiotic consumption volumes, but our analysis indicates that heavy metal use in feed and disinfectant practices should be equally regulated. Microbiome engineering, including defined consortia of next-generation probiotics and phage cocktails targeting ARGcarrying strains, offers a pathway toward precision interventions. Surveillance harmonization across human, animal, and environmental sectors remains a critical unmet need, as does international regulatory coordination to prevent AMR spillover across borders. Collectively, this review provides a network-integrated framework linking HGT mechanisms, co-selection pressures, and interventions across livestock and companion animals. It also critically exposes over-reliance on descriptive metagenomic correlations and proposes specific testable priorities for immediate action under the One Health paradigm.
